# Current Practice Patterns and Educational Needs of the ESCMID Study Group for Infections in Compromised Hots

**DOI:** 10.1111/tid.70076

**Published:** 2025-07-08

**Authors:** Maddalena Giannella, Daniele Riccucci, Renato Pascale, Elisa Cordero, Nicolas J. Mueller, Monica Slavin, Michael Ison, the ECCMID study groups on Invasive Fungal Infection and Infection in Immunocompromised Hosts

**Affiliations:** ^1^ Department of Medical and Surgical Sciences Alma Mater Studiorum University of Bologna Bologna Italy; ^2^ Infectious Diseases Unit IRCCS Azienda Ospedaliero‐Universitaria di Bologna Bologna Italy; ^3^ European Society of Clinical Microbiology and Infectious Diseases Study Group for Infections in Compromised Hosts (ESCICH/ESCMID) Basel Switzerland; ^4^ Department of Medicine School of Medicine University of Seville. Clinical Unit of Infectious Diseases and Microbiology. Virgen del Rocío University Hospital. Institute of Biomedicine of Seville (IBiS). CIBER in Infectious Diseases Seville Spain; ^5^ Department of Infectious Diseases and Hospital Epidemiology University Hospital Zurich and University Zurich Zurich Switzerland; ^6^ National Centre for Infections in Cancer Peter MacCallum Cancer Centre and Sir Peter MacCallum Department of Oncology University of Melbourne Melbourne Victoria Australia; ^7^ Department of Infectious Diseases University of Melbourne Melbourne Victoria Australia; ^8^ National Institutes of Health Bethesda Maryland USA

**Keywords:** clinical microbiology, educational, ESGICH, immunocompromised host, infectious disease, research networks, survey

## Abstract

**Background:**

The ESCMID Study Group for Infection in Compromised Hosts (ESGICH) conducted a survey to assess its members' demographics, clinical focus, training pathways, research activities, and educational needs. The primary objective was identifying the current expertise and challenges professionals face in immunocompromised host infectious diseases (ICH‐ID) and determining how ESGICH can better support their clinical and research endeavors.

**Methods:**

A structured questionnaire was distributed to ESGICH members by email and was posted on X to collect information on work settings, patient populations, training, collaborative networks, research involvement, and educational experiences. The survey also assessed interest in future educational initiatives, including certification programs and targeted training opportunities.

**Results:**

Overall, 119 colleagues participated in the survey, with the majority being members of ESGICH, which had approximately 230 participants, yielding a response rate of 52%. Most of the respondents were from Europe and noted significant involvement in ICH‐ID clinical care and research. Many respondents provide care for transplant recipients and haemato‐oncology patients, with varying levels of institutional support, and often had clinical responsibility beyond the ICH‐ID population. Training in ICH‐ID is inconsistent, with many participants expressing a need for more structured training pathways. Research engagement was high, though support structures varied. Participants identified key educational gaps and expressed interest in webinars, in‐person meetings, and certification programs.

**Conclusion:**

The findings highlight the need for ESGICH to enhance educational opportunities, strengthen research networks, and advocate for standardized ICH‐ID training. Addressing these gaps will improve professional development and ultimately enhance patient care for ICHs.

## Graphical abstract

The ESGICH survey highlights variability in training and support for Immunocompromised Host Infectious Diseases (ICH‐ID). Respondents expressed strong interest in structured education, research collaboration, and certification, underscoring the need for targeted initiatives to strengthen expertise, improve patient care, and define ICH‐ID as a recognized clinical and academic discipline.

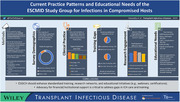

**Abbreviations**
CAR‐T: Chimeric antigen receptor T‐cell therapy
CM: Clinical microbiology
ESCMID: European Society of Clinical Microbiology and Infectious Diseases
ESGICH: Study Group for Infections in Immunocompromised Hosts
HSCT: Hematopoietic stem‐cell transplantation
ICHs: Immunocompromised hosts
ID: Infectious diseases
MDR: Multidrug‐resistant

## 1 Introduction

The prevalence of immunocompromised hosts (ICHs) in the population is increasing, now estimated to be up to 10%, mainly driven by the rising use of immune‐modulating therapies and the aging population [1]. Thus, care decentralization of ICHs beyond specialized referral centers is enlarging. In this framework, the management of infections in this expanding and diverse group has become increasingly complex due to the growing number of immunosuppressive regimens, the emergence of multidrug‐resistant (MDR) infections, and variability in access to specialized expertise [2]. Despite these challenges, infectious diseases (ID) and clinical microbiology (CM) training programs have not kept pace with the rapid evolution of the field, leading to gaps in expertise and inconsistencies in patient management. Indeed, ID/CM clinicians focusing on ICHs operate in diverse clinical settings with varying educational pathways. However, no prior survey had systematically assessed these landscapes exploring potential needs and areas for improvement.

To address these issues, in 2023, the Executive Committee members for the European Society of Clinical Microbiology and Infectious Diseases (ESCMID) Study Group for Infections in Immunocompromised Hosts (ESGICH) designed a survey to characterize the demographic background, clinical practice environment, career pathways, research engagement, and educational needs of ESGICH members.

The results of this survey will help shape future initiatives aimed at improving education, collaboration, and research opportunities for clinicians managing infections in immunocompromised populations.

## 2 Material and Methods

### 2.1 Study Design

An exploratory cross‐sectional international survey was developed and validated by the ESGICH Executive Committee. The survey was distributed to ESGICH members via email, and a reminder was later included in the study group's periodic newsletter. In addition, it was shared through an X (formerly known as Twitter) account. Participants were also asked to share this survey with non‐member collaborators.

The survey was open from February 6, 2024 to March 19, 2024.

### 2.2 Survey Instrument

A 48‐item questionnaire was drawn. The survey (Supporting Information 1) was composed of four parts:

- Respondent's working environment with a focus on the types of immunocompromised populations attended, the proportion of time dedicated to ICHs, and center transplant volume/characteristics;
- Career pathway to the current position and specialized training;
- Research involvement and focus;
- Educational needs and interest in future educational initiatives.

Responses were gathered anonymously. An optional section was included to allow the participant to leave personal contact details in case more detailed information was required and to suggest future research questions to the group.

## 3 Results

### 3.1 Respondents' Characteristics

A total of 119 respondents participated in the survey, the majority of them were ESGICH members. At the time of survey distribution, ESGICH had approximately 230 members. Thus, the response rate was around 52%. Most responses came from European individuals (67%, 80/119) and a total of 30 countries were represented [including Italy (15/119, 13%), Spain (14/119, 12%), the United States of America (9/119, 7.5%), Australia (7/119, 6%), India, Switzerland, Turkey, United Kingdom (6/119, 5% each), and Portugal (5/119, 4%)].

Most respondents (105/114, 92%) cared for adults (75/114, 66%) or adult and pediatric population (30/114, 26%), with a minority focusing only on pediatrics (9/114, 8%). Correspondingly, most participants currently hold an appointment as ID specialists (103/119, 86.5%), with the rest being clinical microbiologists (CM) (11/119, 9%), or other specialties. About 38% (40/105) of respondents reported completing their training within the last ten years, and 52/112 (46%) started their current appointment within the previous ten years.

The majority reported having both an inpatient consultation service (107/112, 95%) and an outpatient clinic (90/113, 80%) dedicated to immunocompromised patients. The main specific immunocompromised populations attended included onco‐hematological patients (100/119, 84.0%), solid organ transplant patients (82/119, 68.9%), and patients with primary immunodeficiency (35/119, 29.4%). A detailed description is reported in Table 1.

**TABLE 1:** Center activity and volume.

	Respondents Reporting the Category	Volume of Activity (procedures per year) *N* (% in the category)				
Category	*N* (% on the overall of respondents)	≤10	11–25	26–50	51–100	>100
HSCT	71 (59.7%)	2 (2.9%)	6 (8.6%)	15 (21.4%)	18 (25.7%)	29 (41.4%)
CAR‐T	49 (41.1%)	14 (28.6%)	7 (14.3%)	17 (34.7%)	8 (16.3%)	3 (6.1%)
Solid organ transplant						
Liver transplant	48 (40.3%)	3 (6.3%)	6 (12.5%)	10 (20.8%)	18 (37.5%)	11 (22.9%)
Kidney transplant	72 (60.5%)	4 (5.6%)	5 (6.9%)	16 (22.2%)	17 (23.6%)	30 (41.7%)
Small bowel transplant	8 (6.7%)	5 (62.5%)	3 (37.5%)	—	—	—
Pancreas transplant	23 (19.3%)	16 (69.6%)	5 (21.7%)	2 (8.7%)	—	—
Heart transplant	35 (29.4%)	5 (14.3%)	21 (60.0%)	7 (20.0%)	2 (5.7%)	—
Lung transplant	29 (24.4%)	9 (31.0%)	6 (20.7%)	8 (27.6%)	3 (10.3%)	3 (10.3%)
Solid tumor	92 (77.3%)	NA	NA	NA	NA	NA
Primary immunodeficiency	35 (29.4%)	NA	NA	NA	NA	NA

Abbreviations: CAR‐T, chimeric antigen receptor T‐cell therapy; HSCT, hematopoietic stem‐cell transplantation.

Thirty‐eight percent of recipients (45/119) reported focusing their clinical care on an immunocompromised population for a proportion higher than 80% of their time, with a quarter of respondents (29/119, 24%) reporting less than 50% of their work time dedicated to ICHs. Respondents who focused on ICHs the entire period of their appointment stated being mostly supported by less than five other colleagues with the same focus in the majority of centers (68/108, 62.9%).

Collaboration with other teams is a hallmark of caring for infections in ICHs. Forty‐nine percent of participants reported having regular combined meetings with the transplant/oncology teams at least once per week (40/82), 20/82 twice per week (24%), and 21/82 (26%) thrice per week or more. More than half also reported conducting ward rounds or sit‐down rounds combined with the transplant/oncology team twice per week or more (51/71, 72%). The majority of respondents (99/114, 87%) reported not receiving fixed funding from the transplant/oncology group or hospital for their activity focused on ICHs.

As for collaboration networks, 83% (95/119) of respondents reported having a group of colleagues outside their institution with whom they could discuss challenging cases. Sixty‐two percent (74/119) also actively participate in activities of a national or regional group focused on immunocompromised or transplant ID.

### 3.2 Center Activity and Volume

Details about the characteristics of the respondent centers and the volume of activity are reported in Table 1. Patients with solid cancer, kidney transplant, hematopoietic cell transplant, and liver transplant were prevalent, with a variable volume of transplant activity per center.

### 3.3 Pathway to the Current Position

**FIGURE 1:**
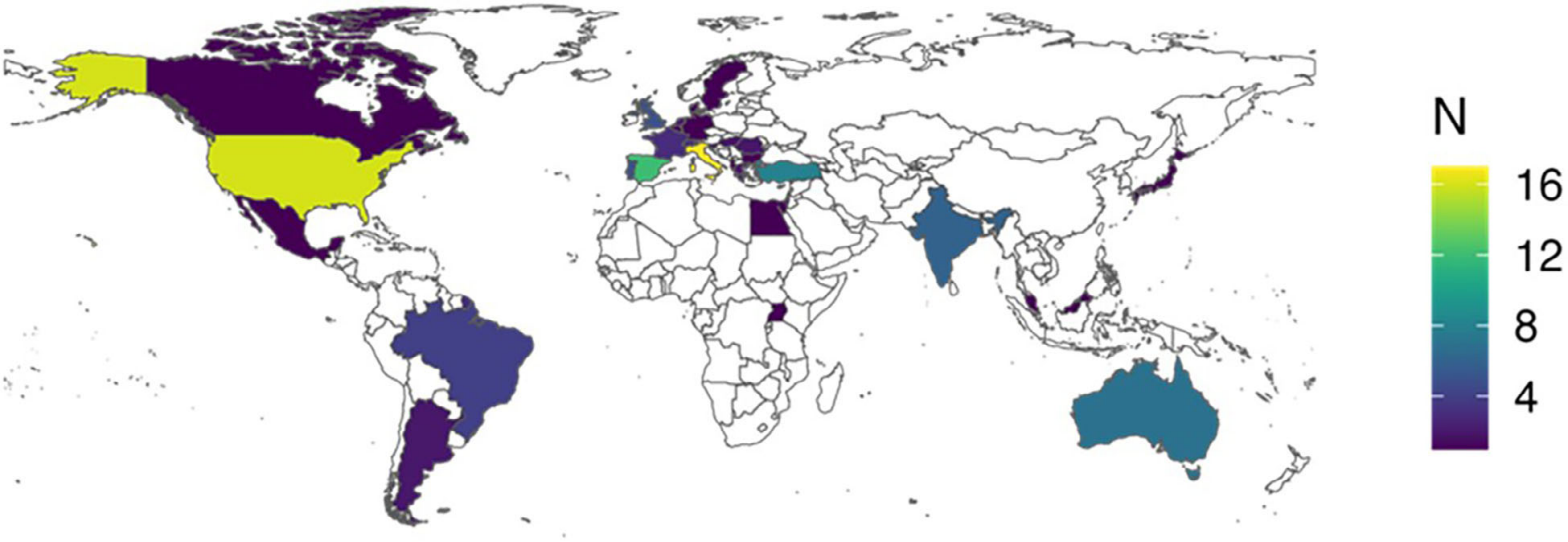
Country of training.

Respondents completed their general (mostly ID or CM) training in 31 different countries, mainly Italy (17/119, 14%), the USA (15/119, 13%), Spain (12/119, 10%), Switzerland (8/119, 7%), and Australia (7/119, 6%), with a median of 5 years of training before their first job (see Figure 1). About 43% (49/113) of respondents reported having received additional training focused on ICHs, but for less than 10 months in 20/41 (48.7%) cases. Most would have liked additional training with this focus (51/58, 87.9%).

In addition, 68% (77/114) of respondents noted that in the institution in which they practice an ID/CM program is active lasting at least 35 months for most centers (41/71, 57.7%), but with less than five months focused on the management of infections in immunocompromised/transplant patients. However, most respondents (75/115, 65%) believed there are opportunities to receive dedicated training on infections in ICHs in their country. In this regard, 81/115 (70%) of respondents stated having the possibility to either host trainees from other programs or to move to other centers for a dedicated rotation in this field. However, respondents noted that few people utilized this opportunity for additional ICH ID training at their center with 59/109 (57.2%) reporting less than 5 trainees in the past 5 years spending ≥ 1 month.

Over half of the participants would have the opportunity to host a trainee for clinical rotations (73/95, 77%) but without funds from their institutions to do so (63/71, 88%) and highlighted that financial support would help this process (51/70, 73%).

### 3.4 Research Experience

Sixty‐three percent (73/115) of respondents reported enrolling immunocompromised or transplant patients in clinical trials and 75% (86/114) had contributed data as part of patient registries in the last two years. Sixty‐six percent (76/115) had participated in translational research focused on this population and 56.5% (65/115) participated in conducting basic science or bench research focused on infections in ICHs in the last two years. About a quarter (32/114, 28%) participated in an official ESGICH study or registry within the previous five years.

Transplant centers, hematology, or oncology units of the respondents support research in this field in about half of the cases (59/119, 49%), usually with access to services (study coordination, regulatory coordination, or data analysis) and, less commonly, with financial support (12/119, 10%). Most respondents (80/115, 70%) do not believe there is sufficient support in their institution for the research activities they are conducting.

### 3.5 Educational Needs

Around 70% (80/115) of respondents attended at least one ESCMID conference and participated in at least one session focused on immunocompromised/transplant ID/CM. Still, the majority felt that more content at these meetings was needed (54/94, 57.4%). Thirty‐seven percent (43/114) of respondents participated in an ESGICH‐sponsored webinar in the previous two years; all of them found it valuable and reported learning something new they could apply in their practice.

The main areas of interest to cover with educational activities were multi‐drug‐resistant bacteria (75/119, 63.0%), novel antifungal therapy (74/119, 62.2%), CAR‐T infectious complications (74/119, 62.2%), CMV (73/119, 61.3%), febrile neutropenia (68/119, 57.1%), donor‐derived infections (61/119, 51.3%), antimicrobial stewardship (60/119, 50.4%), vaccines (55/119, 46.2%), and respiratory viruses (55/119, 46.2%); full list of topics is reported in Table 2.

**TABLE 2:** Research questions respondents think ESGICH should consider.

**Viral**
Viral infections in SOT: CMV, EBV, HHV8, HEV Long/persistent Covid 19 in hematological patients
**Bacterial**
MDR/XDR Gram negative infection treatment in SOT / BSI in SOT The role of microbiome modulation (incl. FMT) for malignant hematological diseases Shorter antibiotic course for immunosuppressed
**Fungal**
Endemic fungal infections in SOT / Failure of antifungal in resource limited countries *Pneumocystis jirovecii* prophylaxis in some groups of ICHs
**Immunology and Prevention**
Antimicrobial stewardship The "net state” of immunosuppression AI/ML‐based predictors for risk of infections Antimicrobial and antifungal prophylaxis in liver transplant patients When to suspect an inborn error of immunity in adults and how to conduct an investigation (algorithms/guidelines)
**Donor Derived Infections**
Long term outcomes of donation from infected donors
**Diagnostic tools**
New diagnostic tools (PCR) for rapid diagnosis of infections Lung infiltrates in immunosuppressed host: how to differentiate infection from non‐infectious causes
**Other Topics**
MTB in SOT Tropical diseases in SOT Epidemiology of toxoplasmosis

Abbreviations: AI/ML, artificial intelligence, machine learning; BSI, bloodstream infection; CMV, cytomegalovirus; EBV, Epstein–Barr Virus; FMT, fecal microbiota transplantation; HEV, Hepatitis E virus; HHV8, human herpesvirus 8; ICH, immunocompromised hosts; MTB, *Mycobaterium tuberculosis*; PCR, polymerase chain reaction; SOT, solid organ transplant.

Most respondents (98/114, 86%) favored having a certification in Immunocompromised ID like the one available through ESCMID for Antimicrobial Stewardship. Most (86/113, 76%) felt that there were insufficient opportunities for trainees to acquire specific training in this field.

Finally, the overwhelming majority (113/114, 99%) would attend a multi‐day in‐person meeting focused on topics from this field and would encourage their trainees to do the same.

## 4 Discussion

This survey helps provide a clearer picture of the ESGICH members, their training related to the management of ICHs, their current clinical experience, and opportunities for education and research. These data are helpful in understanding regional differences in practice and clinical experience relative to clinicians in other parts of the world. Further, it highlights opportunities to provide education targeted to the needs of the community. Lastly, it highlights the need for more structured approaches to training future generations of ID/CM experts on ICHs.

While the survey had respondents from all continents, the majority of respondents were from outside the United States. This provides an interesting perspective, complementing previous reports that focused on the needs of physicians mainly based in the US and Canada [3, 4]. A significant proportion of respondents dedicate most of their clinical time to ICHs, yet they frequently work in small teams with limited dedicated funding for their activities. Despite these constraints, they support their oncology, hematology, or transplant units, participating in joint meetings with them or even clinical rounds. In addition to this internal collaboration network, they also personally participate in research groups outside their institution, which they use to discuss challenging cases and share knowledge. Similar findings were reported in a previous survey conducted by the American Transplant Society [3]. In this report, which primarily included ID physicians from US centers, most respondents reported dedicating between 75% and 100% of their time to managing infections in transplant recipients, including both solid organ and HSCT patients. The multidisciplinary approach was identified as a key priority. While already a well‐established practice in several ID areas, our results highlight its particular value in the care of vulnerable patients with complex needs and atypical clinical presentations, such as ICHs. [3, 5, 6]. Furthermore, most respondents provided care at relatively low‐volume centers, particularly compared to US providers. These findings raise concerns about potential heterogeneity in resource availability and healthcare system organization.

Research engagement among respondents is high, with many involved in clinical trials, translational research, and patient registries. A substantial proportion of our respondents is also involved in basic and bench research. However, research support, particularly financial, remains limited. This trend has been previously reported, with physicians involved in the care of ICHs often receiving insufficient financial backing, whether in terms of personal salary, administrative assistance, or funding for research activities [3, 4, 7].

There is a wide range of educational experiences, with many respondents seeking additional specialized training in immunocompromised ID. With few exceptions (i.e. Spain) [8], standardized ID/CM training programs are available across most countries [9]. However, dedicated subspecialty training focused on ICHs remains limited in the opinion of our respondents. Similarly, in a survey evaluating transplant infectious disease training programs, fellows reported that less than 25% of their training was specifically focused on infections in immunocompromised patients [9]. Therefore, education is a priority, with participants advocating for more ESCMID conference sessions on infections in ICHs and expressing interest in webinars and certification programs. Our survey confirms the well‐recognized need to train physicians by enhancing both clinical and educational experiences in the care of immunocompromised patients, ideally under the guidance of expert mentors in this field [9].

To date, there is no formal ICH infection track at ESCMID Global but its establishment would make it easier for attendees to identify the relevant sessions and opportunities for networking. ESGICH has recently initiated a webinar series that will focus on topics identified in this survey. The support for an in‐person multi‐day meeting further emphasizes the community's desire for structured learning and networking opportunities. This would provide focused education around the full range of ICH issues and would be highly valuable to both junior and more experienced clinicians. ESGICH is currently working to develop such a meeting in the near future.

Lastly, trainee hosting presents both opportunities and challenges. While many respondents are open to hosting trainees, financial constraints limit the feasibility of such initiatives. Support from ESCMID, ESGICH, or home institutions could significantly enhance access to specialized training opportunities, fostering the development of the next generation of experts in immunocompromised ID.

This survey provides important findings but presents some limitations. First, the relatively small number of participants could affect the generalizability of our results. Second, despite the main way of survey distribution being an email to ESGICH members, it was also posted on X potentially reaching a wider audience and more diverse respondents. The impact of this is not measurable and should be recognized. Furthermore, the risk of selection bias due to voluntary participation should be considered. Nevertheless, the needs expressed by respondents appear consistent with findings previously reported in the literature supporting the value of our findings. In addition, our results reflect a single point in time and may not capture evolving trends in education or clinical practice. However, an updated version of this survey is planned to reassess respondents' needs following the development and expansion of ESGICH's activities.

In conclusion, infections in ICHs are an emerging field that is among the most popular among trainees. The members of ESGICH often practice with smaller teams and modest‐sized centers, highlighting the need for formal opportunities to facilitate collaboration. Trainees need more support to get dedicated experience in this field and providers need additional financial and institutional support to facilitate their careers. ESGICH will continue to advocate for educational and research opportunities for its members informed by the results of this survey.

## Author Contributions

Maddalena Giannella, Elisa Cordero, Nicolas J Mueller, Monica Slavin, and Michael Ison conceived the study. Maddalena Giannella and Michael Ison designed and performed the survey. Renato Pascale performed the analysis of data. Maddalena Giannella, Elisa Cordero, Nicolas J Mueller, Monica Slavin, and Michael Ison evaluated the study results. Maddalena Giannella, Daniele Riccucci, and Renato Pascale wrote the first draft of the manuscript. All the authors reviewed the final version of the manuscript.

## Conflicts of Interest

Michael Ison serves as Editor‐in‐Chief of Transplant Infectious Disease. He was not involved in the editorial assessment or decision regarding this manuscript. All the other authors declare no conflicts of interest.

## Supporting information




**Supporting File**: tid70076‐sup‐0001‐SuppMat.docx
