# Supplementary material for: Current Practice Patterns and Educational Needs of the ESCMID Study Group for Infections in Compromised Hots
Source: Transpl Infect Dis. 2025 Jul 8;27(4):e70076. doi: 10.1111/tid.70076 (PMC12416473; doi:10.1111/tid.70076)
Supplement: Supplementary file 1 — Supporting File: tid70076‐sup‐0001‐SuppMat.docx [file TID-27-e70076-s001.docx]

**Supplementary File 1**

**Current Practice Patterns and Educational Needs of the ESCMID Study Group for**

**Infections in Compromised Hots**

**ESGICH Member Survey**

*Demographics and Practice Setting*

Current work location:

1. What country is your primary work location
2. Do you provide consultative care to: Adults Only, Pediatrics Only, Both Adult and Pediatrics
3. Is your current appointment in (multiple choice): Infectious Diseases, Microbiology, Immunology, Other (Specify)
4. Do you have an inpatient consultation service focused on immunocompromised patients (Y/N)
5. Do you have an outpatient clinic focused on immunocompromised patients (Y/N)
6. Which types of patients do you provide care for: Primary Immunodeficiency, Oncology Patients, Stem Cell Transplant Recipients, CAR-T Recipients, Kidney Transplant, Liver Transplant, Pancreas Transplant, Small Bowl Transplant, Heart Transplant, Lung Transplant, Composite Tissue Transplant (face, arm/leg, abdominal wall), Other (Specify)
7. What is the volume of transplants performed at your center in 2023: Stem Cell Transplant Recipients, CAR-T Recipients, Kidney Transplant, Liver Transplant, Pancreas Transplant, Small Bowl Transplant, Heart Transplant, Lung Transplant
8. What proportion of your clinical care is focused on immunocompromised patients (100%, 80-99.9%, 60-79.9%, 50-59.9%, 30-49.9%, 20-29.9%, 10-19.9%, <10%)
9. Where is your primary appointment: Infectious diseases, Oncology, Transplant, Medicine, Hospital, Other (Specify)
10. What year did you complete your infectious diseases training
11. What year did you start your current job
12. Have you been focused on immunocompromised ID the whole time you have been at your current job: Y/N
13. How many individuals at your current center besides you provide immunocompromised/transplant infectious diseases care? #
14. Do you have a network of colleagues outside your institution that you can discuss challenging cases with? Y/N
15. Is there a national or regional group focused on immunocompromised/transplant infectious diseases that you are a member or actively participate in their activities? Y/N
    1. If Yes: Please provide the name(s) of the group
16. Do you receive fixed funding (not related to consultation care) from the transplant group, oncology group or hospital for your immunocompromised/transplant infectious diseases activity: Y/N
17. Do you have regular combined meetings with the transplant/oncology teams? N/Y
    1. If yes: How many per week
18. Do you conduct ward rounds or sit-down rounds combined with the transplant/oncology teams? Y/N
    1. If yes: How many days per week

*Pathway to Current Position*

1. What country did you do your primary infectious diseases training in:
2. How many years of total infectious diseases training and research did you do prior to your first job: #
3. Did you do any training above and beyond the usual infectious diseases training focused on immunocompromised patients: Y/N
   1. (If Yes to above): Where did you do your immunocompromised/transplant infectious diseases training (Country and Center):
   2. (If Yes to above): How long was your immunocompromised/transplant infectious diseases training in months:
   3. (If No to #3): Would you have liked to have done additional training in immunocompromised/transplant infectious diseases?
4. Do you feel that there are opportunities for trainees to get dedicated training in immunocompromised/transplant infectious diseases in your country? Y/N
5. Does your current hospital/university have an infectious diseases training program? Y/N
   1. (If Yes): How many months of training is required as a minimum?
   2. (If Yes): How much training in immunocompromised/transplant infectious diseases is part of the official infectious diseases training program in months?
   3. (If Yes): Is there an option to do dedicated training in immunocompromised/ transplant infectious diseases? Y/N
6. Do you have the ability to have trainees from other programs join your program or you to have a dedicated experience or rotation focused on immunocompromised/ transplant infectious diseases? Y/N
7. In the past 5 years, how many trainees have you spent ≥1 month with focused on immunocompromised/transplant infectious diseases? #

*Research Interests*

1. In the past 5 years, have you enrolled immunocompromised/transplant patients in clinical trials? Y/N
2. In the past 5 years, have you been involved in translational research focused on immunocompromised/transplant patients? Y/N
3. In the past 2 years, have you been conducted basic science/bench research focused on questions relevant to immunocompromised/transplant patients? Y/N
4. In the past 5 years, have you been contributed cases of immunocompromised/ transplant patients to a registry? Y/N
5. In the past 5 years, have you participated in an official ESGICH study or registry? Y/N
6. Do you receive support from your transplant center/hematology/oncology center or hospital to perform immunocompromised host/transplant infectious diseases research? N/Yes – Financial Support/Yes – Access to Services (study coordinators, regulatory coordinators, data analysts)/Yes – Other Support (Specify)
7. Do you feel you have sufficient support at your center to perform the type of research you want to conduct? Y/N
8. More questions about research here?

*Educational Needs*

1. Have you ever attended an ECCMID Conference in person? Y/N
   1. If Yes: Please check the ones you have attended: 2017 – Vienna, 2018 – Madrid, 2019 – Amsterdam, 2022 – Lisbon, 2023 – Copenhagen
   2. Did you attend sessions focused on immunocompromised/transplant infectious diseases? Y/N
   3. Do you feel the immunocompromised/transplant infectious diseases content is: Too Little, Adequate, Too Much
2. In the last 2 years, have you participated in an ESGICH-sponsored webinar? Y/N
   1. If Yes: Did you find the webinar(s) Valuable? Y/N
   2. Did you feel that you learned something new you could apply to your practice? Y/N
3. We are going to be starting a webinar series once per quarter. What topics would you like to see covered: Vaccines, Antimicrobial Stewardship, Laboratory Diagnostics, CMV, EBV, Respiratory Viruses, Hepatitis, HIV, Multi-Drug Resistant Bacteria, Immunology, New Laboratory Diagnostic Methodology, Fever and Neuropenia, Infectious Complications of CAR-T, Novel Fungal Therapy, Donor-Derived Infections, UTIs, Pneumonia, TB, Non-TB Mycobacteria, Endemic Infections, Other (Specify)
4. If there was a pathway to become certified in Immunocompromised Infectious Diseases, similar to what is available for Antimicrobial Stewardship, would you attempt to become certified? Y/N
5. Do you feel there are enough opportunities for trainees to get dedicated training focused on immunocompromised infectious diseases in your country? Y/N
6. If there was a multi-day, in person meeting focused on infections in immunocompromised hosts, would your encourage your trainees to attend? Y/N
7. If there was a multi-day, in person meeting focused on infections in immunocompromised hosts, would you attend? Y/N
8. Do you have other ideas for us to consider focused on education focused on infections in immunocompromised hosts? FREE TEXT

*Optional Personal Details*

The following are optional questions that will allow us to reach out to you and get some additional granular data. Do you agree to share this data? Y/N (If No, end)

1. Name: REQUIRED
2. Email: REQUIRED
3. Hospital/Transplant Center Affiliation (Name):
4. City of your transplant center/hospital:
5. If a trainee was interested in spending a clinical rotation with you, would you be able to host them? Y/N
   1. How much time (in months) could they spend with you?
   2. If there were external funds (from ESCMID, ESGICH or the trainees center) to support the trainee’s time with you, would you be: More likely to have a trainee with you, less likely to have a trainee with you, or it would not impact my interest in the trainee
   3. Do you have support in your center to pay for the trainee during their time at your center? Y/N
6. Is there a topic you would like to be considered as a speaker for future educational programs? FREE TEXT
7. Are there research questions you think ESGICH should consider looking at? FREE TEXT
